# On the Nature of Fear and Anxiety Triggered by COVID-19

**DOI:** 10.3389/fpsyg.2020.581314

**Published:** 2020-11-09

**Authors:** Carlos M. Coelho, Panrapee Suttiwan, Nikolett Arato, Andras N. Zsido

**Affiliations:** ^1^School of Psychology, ISMAI University Institute of Maia, Maia, Portugal; ^2^School of Health of Porto Polytechnic, Psychosocial Rehabilitation Lab, Center for Rehabilitation Research, Porto, Portugal; ^3^Department of Psychology, Faculty of Psychology, Chulalongkorn University, Bangkok, Thailand; ^4^Institute of Psychology, University of Pécs, Pécs, Hungary

**Keywords:** coronavirus disease 2019, anxiety, fear of the unknown, illness anxiety disorder, posttraumatic stress disorder, isolation, disgust sensitivity, media coverage

## Abstract

Emergencies that occur during natural disasters, such as avalanches, earthquakes, and floods, tend to be sudden, unexpected, and ephemeral and recruit defensive responses, similar to the ones recruited when faced with dangerous animals. Defensive behaviors are triggered by activity in survival circuits that detects imminent threats and fear is the conscious emotion of that follows immediately. But this particular threat (COVID-19) is useable and mysterious, triggering anxieties much more than fear. We conducted a literature search on May 1, 2020 in Google Scholar, PsychInfo, and PubMed with search terms related to COVID-19 fears and found 28 relevant articles. We categorized the papers into six groups based on the content and implications: fear of the unknown, social isolation, hypochondriasis, disgust, information-driven fears, and compliance. Considering the nature of fear and anxiety, combined with the characteristics of the present COVID-19 situation, we contemplate that physicians and other health care workers of several specialties, as well as police officers, fire-fighters, and rescue personnel, and first responders might be more able to deal with COVID-19 if they have (a) some tolerance of the unknown, (b) low illness anxiety disorder, (c) tolerance to social isolation; (d) low levels of disgust sensitivity; (e) be granted financial support, (f) have priority if needed medical assistance (g) use caution relatively to the COVID-19 media coverage and (h) be trained to have high levels of efficacy. Possibilities for preventive and therapeutic interventions that can help both health care personnel and the general population are also discussed.

## Introduction

CO stands for corona, VI for virus, D for disease, and 19 designates the year it was discovered. Coronavirus disease 2019 (COVID-19) is caused by the 2019 novel coronavirus (SARS-CoV-2) of probable Pangolin origin (Zhang et al., 2020) with the potential to cause severe respiratory tract infection among infected humans (Chen et al., 2020) and is commonly transmitted from person to person *via* aerosol and droplet contamination.

We are now amidst a current global pandemic declared March 11, 2020, that started in Hubei province of China in late December 2019 and in Europe in February 2020. This pandemic disrupts the lives of people across the world due to its rapid spread, high mortality rate, the toll on health care systems, and devastating economic impact (Callaway et al., 2020). Its spread has been exponential being now in most world countries and becoming an emergent global challenge with over 11.5 million confirmed cases, about 540,000 confirmed deaths as of July 8, 2020. SARS-CoV-2, has been spreading and led to diverse clinical symptoms (COVID-19) including but not limited to cough, high fever, fatigue, and shortness of breath. Especially older individuals and/or those with other medical conditions are at risk of developing severe respiratory problems in the course of COVID-19. In such situations, the disease may progress to multi-organ failure, pneumonia, and death (Centers for Disease Control and Prevention, 2020; World Health Organization, 2020). Here, we alert for the need to create some tranquility in the media and political positions. If people are allowed to know more about what we know and do not know about the complexity of what we are dealing with, a wrong sense of understanding the causal processes underlying policies will contribute to further political polarization (Fernbach et al., 2013) and this, in turn, will enhance more fear and suspicion (Brooks et al., 2020). Presented with such a high infection rate and mortality, individuals are disquieting. Fear can strengthen the damage of the disease, leading individuals to not think rationally when reacting to COVID-19 (Ahorsu et al., 2020). On the opposite, insufficient fear can result in harm for individuals and society (e.g., ignoring government measures or reckless policies; Mertens et al., 2020). Next, we discuss the main features associated with the potential worries and fears related to COVID-19 with the final aim of speculating about some personality characteristics likely more resilient to deal with infected people.

## Materials and Methods

We used a two-stage systematic approach to identify articles that examined the effect of emotional arousal on visual search performance. The initial search was conducted on May 1, 2020 in Google Scholar, PsychInfo (journal article subdatabase), and PubMed with search terms [(“fear” OR “phobia”) AND (“COVID” OR “COVID-19” OR “coronavirus” OR “SARS-CoV-2” OR “SARS coronavirus”)]. After the initial search, we removed duplicates and examined the resulting articles’ references to ensure all relevant papers were included. There were no exclusion criteria for the type of participant sample (e.g., clinical). The search resulted in 28 peer-reviewed papers. We categorized the papers into six groups based on the content and implications: fear of the unknown, social isolation, hypochondriasis, disgust, information-driven fears, and compliance. Table 1 shows the included studies. In the next section, we are going to discuss these factors with regard to previous results regarding other pandemics and theories of fear.

**Table 1:** Studies included in this review were grouped based on the type of fear they are tapping into.

Group	Study
Fear of the unknown	
	Mertens et al., 2020 Satici et al., 2020b
Social isolation	
	Bradbury-Jones and Isham, 2020
	Casale and Flett, 2020
	King et al., 2020
	Lin, 2020
	Mertens et al., 2020
	Thombs et al., 2020
	Yang et al., 2020
Hypochondriasis	
	Akgün et al., 2020
	Asmundson and Taylor, 2020
	Banerjee, 2020
	McKay et al., 2020
	Rajkumar, 2020
	Schimmenti et al., 2020
	Thombs et al., 2020
	Vanni et al., 2020
	Wong et al., 2020
Disgust	
	Brooks et al., 2020
	McKay et al., 2020
	Mota et al., 2020
	Satici et al., 2020b
	Troisi, 2020
Information-driven	
	Ali, 2020
	Asmundson and Taylor, 2020
	Erku et al., 2020
	Landau-Wells and Saxe, 2020
	Sefidbakht et al., 2020
Compliance	
	Brooks et al., 2020
	Fernandez, 2020
	Harper et al., 2020
	Jørgensen et al., 2020
	Mertens et al., 2020
	Olesen et al., 2020
	Presti et al., 2020

## Results

### COVID-19 Comprises Multiple Fears

Emergencies that occur during natural disasters, such as avalanches, earthquakes, floods, and hail, and human-made disasters, such as a building collapse, air disasters, industrial/technological accidents, and fires tend to be sudden, unexpected, and ephemeral. These kinds of threat recruit defensive responses similar to the ones recruited during unexpected personal situations such as when crossing a road, riding a bicycle or driving a car, or when faced with dangerous animals or people (Zsido et al., 2020b). Defensive behaviors are immediate responses (LeDoux, 2012) triggered by activity in survival circuits that detects threats (LeDoux, 2014) and leading to the conscious emotion of fear that follows immediately. But this particular threat (COVID-19) is useable and mysterious even, triggering anxiety much more than fear. Dealing with it requires prolonged coping mechanisms more than immediate defensive reactions.

The current COVID-19 pandemic presents a significant occupational hazard for physicians and other health care workers of several specialties such as those who perform or participate in head and neck region examinations (e.g., Ota and Asada, 2020), dental and oral medicine (Meng et al., 2020), ophthalmologists (e.g., Shabto et al., 2020), etc. Delayed access to hospital care for emergency conditions deriving from workers’ multiple roles adds to the panoply of stressful conditions, affecting many with unrelated problems such as children’s occasional infections, acute onset of chronic conditions, endocrine disorders (e.g., diabetes), or surgical needs (e.g., appendicitis; Lazzerini et al., 2020).

People worry that individual and societal economic resources might be scarce or unable to recover any time soon (Thombs et al., 2020). Societal safety measures such as the lockdowns designed to prevent the spreading of infections, if too prolonged or strict, can disrupt the economy and bring unemployment. The economic consequences of the COVID-19 pandemic also have a psychological impact on individuals worldwide due to the loss of jobs of millions of individuals told to remain in their houses, when unable to work from home (Pakpour and Griffiths, 2020). This is leading to a financial crisis and recession, and an overall suicide rate increment (Mamun and Ullah, 2020).

The COVID-19 pandemic formed a serious multi-etiological global mental health challenge influencing every aspect of life and disrupting the social fabric. COVID-19 is a situation able to bring about several fears (e.g., contamination, future, financial instability, xenophobia, and agoraphobia, etc.) and to trigger elements related to anxiety and fear (similar to specific phobias). Fear is usually avoided, but like pain or hunger, it can be adaptive to deal with imminent threats. Anxiety can also be adaptive to deal with potential threats, but when not well calibrated to the actual threat, it can be deleterious, both at the individual and societal levels (Mertens et al., 2020). An increased level of concern does not necessarily lead to intention to self-isolate – indeed, the opposite may be true in some cases (Bacon and Corr, 2020). It is imperative to understand how personality influences the way people’s reactions differ in response to the present situation.

### Fear of the Unknown and Intolerance of Uncertainty

The fear or anxiety can be brought about both by knowing or having more information and by fear of the unknown related to the virus. In fact, an uncertain and continuous threat can become chronic and burdensome (Mertens et al., 2020). With many infected people being asymptomatic, reports and calculations on the fatality rate are impossible to perform accurately, and there is no way for a person to know if the other next to him is infected or not, adding more uncertainty to the situation. Intolerance to uncertainty is related to when the unknown is perceived intensely resulting in anxiety (Fergus, 2013). Fear of the unknown appears to be a fundamental fear and is a core component of anxiety (Gallagher et al., 2014; Carleton, 2016). COVID-19 related fears recruit not only fear of the unknown but also the anxiety that accompanies situations that are unpredictable and uncontrollable. So the fear at this undetectable threat is easily learned, irrespective of the probability of its occurrence. Accordingly, a study on COVID-19 (Satici et al., 2020b) corroborated that the inability to tolerate uncertainty is related to fear of COVID-19 *via* rumination, and this affected well-being due to the prominent focus on negative emotions. No matter how much training a person endures, they will likely need some tolerance to uncertainty, particularly at this stage.

### Social Isolation and Social Support

There is a worry that isolation and movement restrictions will be long-lasting with a heavy toll on mental health and well-being, social functioning, and work (Thombs et al., 2020). As the fear of contagion and proximity to others (Lin, 2020) is high, many millions of people have begun working remotely and billions are quarantined or isolated at their own homes, schools and universities canceled face-to-face classes, and restaurants, bars, gyms, and other gathering places in many countries have closed (Casale and Flett, 2020). Still, Mertens et al. (2020) found concerns for others’ to be the most often indicated concern. Stressful situations increase the need for social support and to affiliate with others; people who typically are highly focused on their interpersonal needs will suffer more with the current pandemic and imposed conditions of social isolation (Casale and Flett, 2020). The perceived discrepancy between the desired and actual quality of social relationships – loneliness – can have serious mental and physical health effects, threatens the sense of safety and well-being (Stickley et al., 2016; Leigh-Hunt et al., 2017; Holt-Lunstad, 2018) and is linked to hypochondriasis (Brink and Niemeyer, 1993) and obsessive–compulsive symptoms (Timpano et al., 2014). The lockdown can also facilitate problematic behaviors such as online gaming (King et al., 2020), domestic violence (Bradbury-Jones and Isham, 2020) as well as stigma and xenophobia (e.g., Yang et al., 2020). Contrary to most doctors, nurses, police officers, fire-fighters, etc. that are often working in teams and very hardly ever alone, the present situation requires people to isolate, and this alone can be too hard to take for some professionals in the frontline.

### Hypochondriasis

COVID-19 can carry many fears and worries associated. Schimmenti et al. (2020) mention among others, the fear of body symptoms and their possible meanings. Hypochondriasis is named as illness anxiety disorder in the DSM-5 manual (American Psychiatric Association, 2013) and can be likely related to hospital emergency flow of people who misinterpret their bodily sensations as signs of potential infection (Asmundson and Taylor, 2020). Individuals prone to monitor physical sensations would benefit from education regarding the potential for false alarms regarding these interpretations by decreasing anxiety (McKay et al., 2020). Anxiety might lead to obsessive use of medications like hydroxychloroquine, which has recently emerged in guidelines for COVID-19 (Banerjee, 2020).

COVID-19 is also related to the worry that health care systems may be overrun and that adequate medical care will not be available for all those affected (Thombs et al., 2020) or others that have different health problems. Yet, many patients in need of medical care avoid hospitals (Wong et al., 2020). Some patients refuse surgical treatment due to fear of COVID-19 contagion even at the risk of survival (Vanni et al., 2020) and patients’ fear and suffering among intensive are now magnified. Many patients are unable to communicate consistently if at all, causing fear of abandonment, feelings of isolation, psychological suffering that can and should be mitigated with ongoing, bi-directional communication strategies (see Akgün et al., 2020).

Also related to fear of contamination is obsessive-compulsive disorder (OCD). OCD has distinct dimensions, namely, (a) fear of contamination and cleaning compulsions, (b) obsessions of repugnant or taboo nature and checking compulsions, (c) obsessions and compulsions related to symmetry, and (d) hoarding. Researchers suggest that there may be close links between some dimensions of OCD and behaviors that evolved to protect our ancestors from infectious diseases (Rajkumar, 2020). Worldwide there have been reports of increased symptoms, distress, and concern about OCD and also hoarding disorder (Banerjee, 2020) related to COVID-19.

### Disgust

Disgust is also related to previous fears such as illness anxiety disorder and OCD. Mota et al. (2020) found the pandemic affected people’s dreams, reflecting mainly fear of contagion, and important changes in daily habits related to contamination and cleanness. Worries of personal infection or infection of friends and family members are common among people exposed to any infectious disease outbreak (Brooks et al., 2020). A paper from Troisi (2020) sums up very well how fear of COVID-19 infection is biologically predisposed, likely to reflect a biologically predisposed form of learning. As stimuli that trigger disgust are also often potential vehicles of infection, such as feces, rotten flesh or food, and body fluids such as blood, sneezes, cough, vomit, or bad breath. Similarly to other fears, the selection set a low threshold for disgust, being triggered by innocuous stimuli, in a brain prewired to over-respond (Nesse, 2005) and fear harmless stimuli, such as congenital malformations (Troisi, 2020). Therefore, being in close physical proximity to those people categorize as in a potential risk group can result in maladaptive psychological consequences – e.g., anxiety or depressive mood – during epidemics (Satici et al., 2020b). This is supported by previous research showing that disgust domains (propensity and sensitivity) positively predicted contamination fear (Olatunji et al., 2004; Cisler et al., 2007) and behavioral avoidance in contamination fear (Deacon and Olatunji, 2007); especially regarding blood–injection–injury type fears (Sawchuk et al., 2000). Relatively to COVID-19, both disgust propensity – that is, the likelihood to experience disgust in the presence of common disgust elicitors – and disgust sensitivity – that is, the degree to which one interprets physical sensations as resulting from disgust and the potential of a contaminant being present – to predict fear of contracting COVID-19 (McKay et al., 2020).

### Political and Information-Driven Fears

The COVID-19 pandemic brought an extraordinary challenge to policymakers as well. In fact, a connection has been shown between individual differences for political organization and sensitivity to threats (Landau-Wells and Saxe, 2020). Further, it is well-known that people regularly hold extreme positions about complex policies regarding which they know less about than they think they do (see e.g., Carpini and Keeter, 1996; Rozenblit and Keil, 2002; Fernbach et al., 2019). This erroneous idea of understanding the causal processes underlying policies contributes to political polarization (Fernbach et al., 2013). Extreme ideologies are characterized by a relatively simplistic, black-and-white perception of the social world, overconfidence, and intolerance (van Prooijen and Krouwel, 2019), leading to beliefs in simple solutions to a complex crisis (e.g., van Prooijen et al., 2018).

This way of thinking enters in direct clash with scientific thinking, always researching, confirming, exploring, and changing. The need for rapid study and research into COVID-19 has stirred the social, political, and scientific world. For example, in February 2020, health authorities advised people that masks and gloves were not indispensable for avoiding infection in healthy people (Asmundson and Taylor, 2020) but since policies changed (in May) and currently people are required to use masks when inside public spaces. As uncertainty and fear are particularly strong among the political extremes, the present rapid changes mixed with misinformation and fear can bring about a cycle of fear and mistrust amidst the COVID-19 pandemic.

And as if all this were not enough, we are also surrounded by rumors and conspiracy theories as well as geopolitical strategies and counterstrategies at a global level, eventually affecting how the outbreak is managed (Ali, 2020). There is a proliferation of fake medicines, fake news, and medication misinformation surrounding COVID-19 (Erku et al., 2020). For example, the belief that consumption of alcohol can be beneficial in preventing the COVID-19 infection leads to an outbreak of methanol poisoning in Iran (Sefidbakht et al., 2020).

### Fear, Efficacy, and Compliance

A recent study (Harper et al., 2020) using Ahorsu et al. (2020) Fear of COVID-19 Scale found that perceiving COVID-19 threat as severe was positively associated with preventive behaviors, suggesting that perceived threat can be a motivational factor to smooth the progress of prevention, being a normal and functional response within the present context. Hence, if fear can trigger safety behaviors in some people and might be able to mitigate contamination, officials should take measures to ensure that to tell people what is happening and why and provide clear communication reinforcing the sense of altruism (Brooks et al., 2020). The pandemic disease causes patients, health professionals, and the general public to endure an overpowering psychological pressure. Not only the disease itself and the losses it imposes are frightening and costly but also too are the social regulations and behavioral adjustments required combating the disease (Presti et al., 2020). Mertens et al. (2020) suggest that media communication should be clear and unambiguous to reduce uncertainty, without sensationalism or disturbing images.

Further, they also argue that media communication should avoid inducing more fear because that likely will not promote behavioral change (see also Peters et al., 2013). Moreover, feelings of anxiety and fear may not predict high levels of protective behavior among the public (Jørgensen et al., 2020). In contrast, authorities can increase compliance by fostering feelings of efficacy, particularly among those who do not feel threatened, promoting compliance without fear (Jørgensen et al., 2020). In sum, people need to know and be trained in the specific protective measures and feel capable of following them (Rippetoe and Rogers, 1987; Jørgensen et al., 2020). This is particularly useful to health care workers, police, fire-fighters, and rescue personnel as they tend already to show lower fear levels compared to the general population.

Fernandez (2020) pinpointed several influential unmet needs related to stress and psychological problems among medical staff stressing and well summarizing our previously mentioned arguments. The factors include insufficient communication, lack of (and also erratic) information, inadequate protective equipment, fear of the unknown and uncertainty, concern about infection leading to self-isolation and thus, vulnerability to stress, anxiety, depression, insomnia, and fear. An approach taking into account fostering feelings of efficacy was initiated in a Danish Hospital (Olesen et al., 2020) by intensive education of all staff and facilitation collaboration between infection prevention and control nurse and a psychologist. Combining psychoeducation in coping strategies toward fear and high level of stress, how to use personal protection equipment (PPE) correctly. The staff became confident of their ability to assess risk behavior when close to patients with COVID-19 and began trusting their knowledge of infection prevention and the correct use of PPE. This approach enhanced rational thinking and fostered a professional attitude (Olesen et al., 2020).

## Conclusion

Research on the psychological reactions to previous epidemics and pandemics suggests that various psychological vulnerability factors may play a role in the extreme anxiety some people might presently manifest. COVID-19 survivors are at risk of developing posttraumatic stress disorder (PTSD) in particular (a) hospitalized individuals, (b) individuals who were not provided healthcare, (c) healthcare workers and other professionals at risk during the pandemic, (d) stigmatized groups, and (e) individuals with mental health problems such as depression, anxiety, and substance misuse disorders, and other severe conditions such as brief reactive psychosis were also reported (Anmella et al., 2020; Sękowski et al., 2020). Individual differences such as intolerance of uncertainty, perceived vulnerability to disease, and anxiety (worry) proneness were stressed by previous research (Taylor, 2019; Asmundson and Taylor, 2020). Similarly, through an online study conducted in March 2020, Mertens et al. (2020) found intolerance of uncertainty, health anxiety, the risk for loved ones, and consulting more information sources (e.g., regular media, social media, and professional media) as independent predictors for the fear of the coronavirus.

There are, of course, possibilities for preventive and therapeutic interventions that can help both health care personnel and the general population. Social support, for instance, has long been posited as a protective factor against the psychological and physiological impacts of exposure to negative life events such as fear and stress. (Cohen and Wills, 1985; Thoits, 1986, 2011; Uchino et al., 2012; Zeidner et al., 2016; Vine et al., 2019). Further, adaptive emotion regulation strategies used to cope with stressors can result in a more positive subjective well-being (Gross and John, 2003; Garnefski and Kraaij, 2006; Mauss et al., 2013; Kraaij and Garnefski, 2019). A recent study (Zsido et al., 2020a) examined how university students coped with the negative mental health effects of the COVID-19 lockdown and found that the most prominent protective factor was positive refocusing, a cognitive emotion regulation strategy that increased mental well-being, reduced depression and anxiety symptoms, loneliness, and problems with sleeping. Regarding therapeutic interventions, the cognitive-behavior therapy approaches may help by focusing on reducing the negative thoughts, worry, and anxiety symptoms potentially leading to excessive fears. Members of the health care personnel and the general population can use self-monitoring to recognize maladaptive patterns in their thoughts and behaviors. Physical exercise and activity along with relaxation, distress tolerance, and acceptance can help cope with these thoughts (Benhamou and Piedra, 2020; Murphy et al., 2020). Proper communication by experts and others can also promote resilience, e.g., by providing a clear, optimistic vision and a realistic plan, taking decisive action, and facilitating open and frequent communication (Kinman et al., 2020; Wu et al., 2020).

Considering the nature of fear and anxiety, combined with the characteristics of the present COVID-19 situation, we contemplate that physicians and other health care workers of several specialties, as well as police officers, fire-fighters, and rescue personnel, and first responders might be more able to deal with COVID-19 if they have (a) some tolerance of the unknown, (b) low illness anxiety disorder, (c) tolerance to social isolation; (d) low levels of disgust sensitivity; (e) be granted financial support, (f) have priority if needed medical assistance; (g) use caution relatively to the COVID-19 media coverage; and (h) be trained to have high levels of efficacy. Ahorsu et al. (2020) Fear of COVID-19 Scale used a sample comprised 717 Iranian participants and there are already Turkish (Satici et al., 2020a), Bengali (Sakib et al., 2020), Arabic (Alyami et al., 2020), Israeli (Bitan et al., 2020), and Italian versions (Soraci et al., 2020). Lee (2020) also created the Coronavirus Anxiety Scale. These scales would likely correlate with the above-mentioned variables. Future research should focus on pointing to protective and risk factors of psychological well-being and also to show what variables predict specific fear and anxiety in such scenarios.

## Author Contributions

Conceptualization: CC. Methodology: CC and AZS. Formal analysis and investigation: AZS, NA, and PS. Writing – original draft preparation and supervision: CC and PS. Writing – review, editing, and resources: AZS and NA. Funding acquisition: CC, PS, NA, and AZS. All authors contributed to the article and approved the submitted version.

### Conflict of Interest

The authors declare that the research was conducted in the absence of any commercial or financial relationships that could be construed as a potential conflict of interest.
